# Women’s Attitude Toward Fertility And Childbearing in Saudi Arabia

**DOI:** 10.1192/j.eurpsy.2024.1733

**Published:** 2024-08-27

**Authors:** A. A. Aloraini, R. A. Alamri, D. S. Alassaf, E. Alenzi, D. A. Alateeq, N. I. Almutlaq, R. H. jahhaf, A. A. Almajhad, S. S. Aloglla

**Affiliations:** 1 College of Medicine, Princess Nourah bint Abdulrahman, riyadh, Saudi Arabia

## Abstract

**Introduction:**

The decline in fertility is one of the major problems worldwide that could affect family structure. Many studies have been conducted to assess attitudes toward fertility and childbearing around the world, but there’s a lack of research about that in Saudi Arabia (SA). The study aims to assess women’s attitudes toward fertility and childbearing in SA and to investigate its association with sociodemographic, medical, and psychological factors.

**Objectives:**

Aim of the Study: The purpose of the study is to assess women’s attitudes toward fertility and childbearing in Saudi Arabia and to investigate its association with sociodemographic, medical and psychological variables.

Specific Objectives:

1. To assess women’s attitudes toward fertility and childbearing in Saudi Arabia.
2. To investigate association between attitudes toward childbearing with sociodemographic characteristics in Saudi Arabia.
3. To investigate association between attitudes toward childbearing with medical and psychiatric history in Saudi Arabia.
4. To investigate association between attitudes toward childbearing with the childbearing preferences in Saudi Arabia.

**Methods:**

This cross-sectional study of a convenient sample of 2172 women in SA in Dec 2022 and Jan 2023. Data were collected through a survey link that contains:1)Sociodemographic data, 2) medical and psychiatric history, 3) childbearing preference and 4) the Arabic version of the Attitudes toward Fertility and Childbearing Scale (AFCS). Data were analyzed by SPSS 25 ;We described the variables in means ± SD or percentage as appropriate. Student’s t-test and ANOVA were performed to analyze differences between the components and background characteristics.

**Results:**

Individuals in the age group of 18-25 years (25.54± 9.08, p<0.001), unmarried (25.23 ± 8.87, p<0.001), and diagnosed with a psychiatric disorder (24.76 ±9.51, p<0.002) scored lower in importance of future of childbearing. In terms of hindrance at present and childbearing preparation, individuals in the age group of 18-25 years (25.66 ± 8.66, p<0.001) (18.53 ± 5.08, p<0.001) respectively, unmarried (25.71± 8.58, p<0.001) (18.46 ± 5.08, p<0.001) respectively, and students (25.92 ± 8.82, p<0.001) (18.55 ± 5.15, p=0.001) respectively were more likely to score high. Participants who had not made a decision about having children (9.36±3.32, p<0.001) scored lower in the female identity domain.

**Conclusions:**

In conclusion, the findings of this study indicate that the younger age group (18-25 years) and those with psychiatric illnesses scored lower in the importance for future of childbearing compared to women of older age group (36-49 years ) and those without psychiatric illnesses. On the other hand, college students showed more concerns related to childbearing hindrance and preparation.

**Disclosure of Interest:**

None Declared

